# Prognosis of IgA nephropathy patient with proteinuria remission by supportive therapy: cohort from screening failed Chinese patients in TESTING study

**DOI:** 10.1080/0886022X.2024.2398826

**Published:** 2024-09-09

**Authors:** Yingman Guo, Sufang Shi, Jinghong Zhao, Caili Wang, Zhangsuo Liu, Shuxia Fu, Nan Chen, Guisen Li, Lihua Wang, Zhaohui Ni, Haitao Zhang, Lingyun Lai, Jicheng Lv, Hong Zhang

**Affiliations:** a Renal Division, Department of Medicine, Peking University First Hospital, Peking University Institute of Nephrology, Key Laboratory of Renal Disease, Ministry of Health of China, Key Laboratory of Chronic Kidney Disease Prevention and Treatment (Peking University), Ministry of Education, Beijing, China; b Department of Nephrology, The Key Laboratory for the Prevention and Treatment of Chronic Kidney Disease of Chongqing, Chongqing Clinical Research Center of Kidney and Urology Diseases, Xinqiao Hospital, Army Medical University (Third Military Medical University), Chongqing, China; c The First Affiliated Hospitals of Baotou Medical College, Inner Mongolia University of Science and Technology, Baotou, China; d Department of Nephrology, the First Affiliated Hospital of Zhengzhou University, Zhengzhou, China; e Department of Nephrology, The Second Hospital, Hebei Medical University, Shijiazhuang, Hebei, China; f Department of Nephrology, Institute of Nephrology, Shanghai Ruijin Hospital, Shanghai Jiao Tong University School of Medicine, Shanghai, China; g Renal Division and Institute of Nephrology, Sichuan Provincial People’s Hospital, School of Medicine, University of Electronic Science and Technology of China, Sichuan, China; h Division of Nephrology, Shanxi Medical University Second Hospital, Shanxi Kidney Disease Institute, Taiyuan, China; i Department of Nephrology, Renji Hospital, School of Medicine, Shanghai Jiao Tong University, Shanghai, China; j National Clinical Research Center of Kidney Diseases, Jinling Hospital, Nanjing University School of Medicine, Nanjing, China; k Division of Nephrology, Huashan Hospital, Fudan University, Shanghai, China

**Keywords:** IgA nephropathy, TESTING study, intensive supportive therapy, screening failed patients

## Abstract

**Background:**

During the run-in phase of the TESTING study, approximately half of patients with IgA nephropathy (IgAN) were excluded due to proteinuria below 1 g/24 h after intensive supportive therapy. The long-term prognosis of these patients needs further investigation.

**Methods:**

112 screening failed patients in the TESTING study from 10 centers in China were enrolled in this retrospective study. The prognosis of 88 patients, who were excluded because of proteinuria below 1 g/24 h, was analyzed by Landmark Kaplan-Meier analysis. The composite kidney endpoint was defined by *a* ≥ 50% reduction in eGFR, ESKD (eGFR <15 mL/min per 1.73 m^2^), chronic dialysis for at least 6 months, or renal transplantation.

**Results:**

In total, 88 patients were excluded due to proteinuria less than 1 g/24 h. During the follow-up, 73/88 (83.0%) patients received renin-angiotensin system blocker. 72/88 (81.8%) had stable proteinuria remission and did not receive immunosuppressive therapy (IST), and 16/88 (18.2%) received IST because of a relapse of proteinuria. Landmark Kaplan–Meier analysis revealed that, the kidney survival from dialysis or composite kidney outcome of these excluded patients with IST was similar to those without IST during the early stages of follow-up (dialysis, before 60 months, *p* = 0.778; composite kidney outcome, before 48 months, *p* = 0.862); whereas the risk for dialysis of patients receiving IST was significantly higher than those without IST after 60 months (OR = 11.3, *p* = 0.03). Similarly, the risk for the composite kidney outcome of patients receiving IST was also significantly higher than those without IST after 48 months (OR = 5.92, *p* = 0.029).

**Conclusions:**

IgAN patients who maintained a persistent remission of proteinuria after intensive supportive therapy had a much better long-term kidney outcome than those who experienced a relapse of proteinuria and needed IST.

## Introduction

IgA nephropathy (IgAN) is the most common primary glomerular disease worldwide [1,2]. Most IgAN patients experience a slowly progressive condition, with approximately 20%–30% of patients progressing to end-stage kidney disease (ESKD) within 10–20 years of diagnosis. Proteinuria has been proven to be one of the most potent predictors of ESKD in IgA nephropathy [3–5]. Kidney Disease Improving Global Outcomes (KDIGO) guidelines recommend that IgAN patients who have proteinuria more than 0.5 g per day should receive treatment with a blocker of the renin–angiotensin system [6–8]. The Therapeutic Effects of STeroids in IgA Nephropathy Global (TESTING) study was designed and aimed to evaluate the effects and safety of steroids in high-risk IgAN patients, who were defined as proteinuria greater than 1.0 g/24 h after receiving intensive supportive treatment for 4–12 weeks according to the KDIGO guidelines [9,10]. TESTING study confirmed the efficacy of steroids in high-risk IgAN patients [11]. However, the long-term prognosis and benefit of steroids therapy of patients who have achieved proteinuria less than 1 g/24 h after at least 12 weeks of intensive supportive treatment, need to be investigated.

During the screening and run-in phase of the TESTING study, about half of participants were excluded because of proteinuria less than 1 g/24 h after intensive supportive therapy. In the present study, we intended to investigate the clinicopathological characteristics and kidney prognosis of these excluded patients because of proteinuria remission by renin-angiotensin system blocker (RASB) therapy alone in the TESTING study in China.

## Methods

### Study design and population

**Figure 1.**
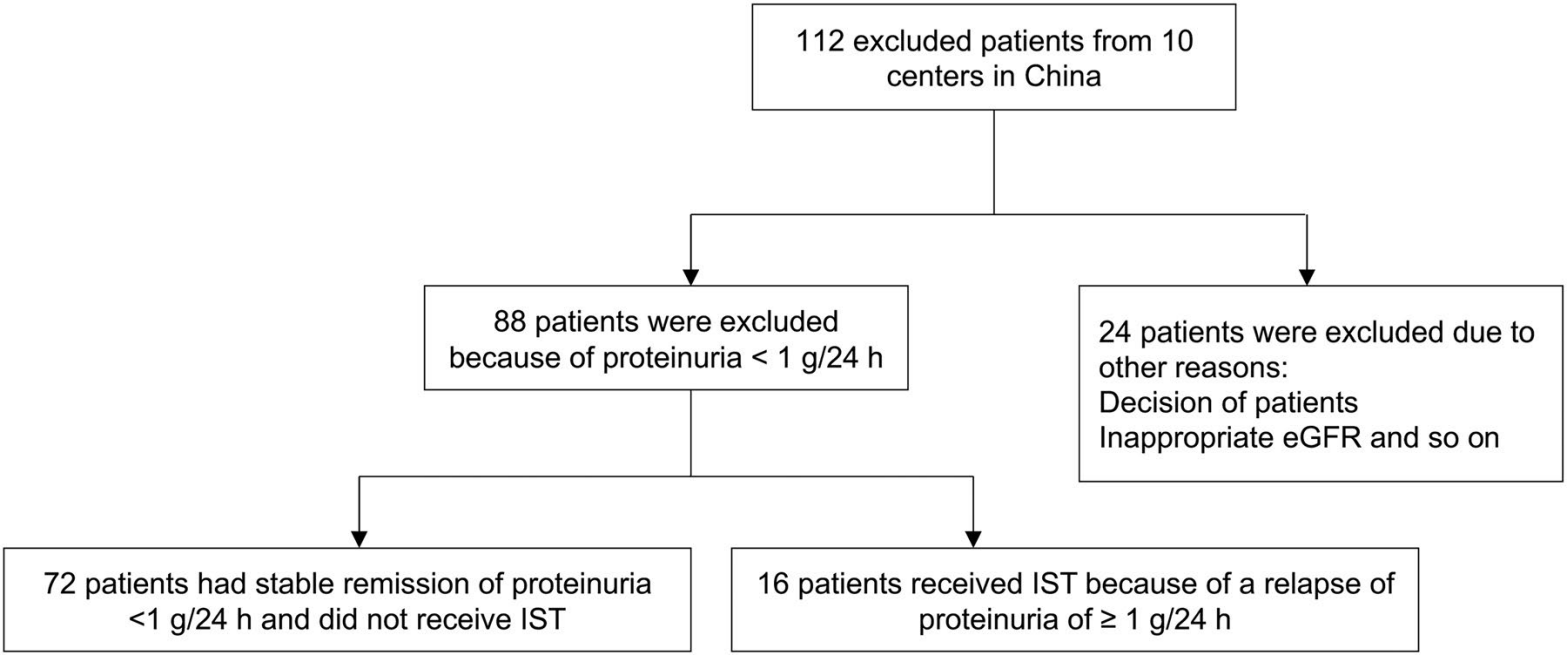
Flow diagram of the study population.

The eligibility criteria for the TESTING study included a diagnosis of primary IgA nephropathy proven by kidney biopsy, an estimated glomerular filtration rate (eGFR) between 20 and 120 mL/min/1.73 m^2^ (calculated using the Chronic Kidney Disease Epidemiology Collaboration formula; modified to 30 to 120 mL/min/1.73 m^2^ for the reduced-dose cohort), and 24-h urinary protein excretion greater than or equal to 1 g per day. Potentially eligible participants underwent a run-in period of up to 12 weeks, to optimize supportive therapy, which included the use of maximal renin-angiotensin system blocker therapy (Ramipril or ARB) for at least 3 months prior to randomization. Participants with proteinuria below 1 g per day during the run-in period were excluded. Between May 2012 and November 2019, a total of 950 potentially eligible participants were screened for the TESTING study and 447 were excluded during the run-in phase, of whom 249 (55.7%) were excluded because of proteinuria less than 1 g/24 h at the end of screening. In the present study, 112 eligible participants who were screened and excluded for the TESTING study during the run-in phase from 10 centers in China were enrolled, including 88/112 (72.1%) patients who were excluded due to proteinuria less than 1 g/24 h, and 24 patients who were excluded because of other reasons (patients’ decision, inappropriate eGFR and so on) (Figure 1). Clinical data, including demographics, serum creatine, eGFR, mean arterial pressure (MAP) and proteinuria during the run-in period as well as eGFR and proteinuria at the end of follow-up, were recorded. Written informed consent was obtained from all participants, and the protocol was reviewed and approved by the Ethics Committee of the Peking University First Hospital (Ethics approval number 2021-245).

### Definitions

Primary IgA nephropathy was defined by the presence of diffuse dominant or codominant mesangial deposits of immunoglobulin A (IgA). Patients secondary to liver disease, lupus or IgA vasculitis (Henoch–Schonlein purpura) were excluded. Proteinuria, MAP, serum creatinine, and eGFR were collected at the time of the run-in period. We estimated the eGFR using the CKD-EPI formula, and the MAP was calculated as diastolic BP + 1/3(systolic BP - diastolic BP). Immunosuppressive therapy (IST) was defined as treatment with corticosteroids or any immunosuppressive agents after the run-in phase regardless of the duration or dose. RASB treatment was defined as the patient’s use of an angiotensin–converting enzyme inhibitor(Ramipril), and/or angiotensin receptor blocker during the subsequent follow-up after being excluded from the TESTING study. The kidney composite endpoint was defined by *a* ≥ 50% reduction in eGFR, ESKD (eGFR <15 mL/min per 1.73 m^2^), chronic dialysis for at least 6 months, or renal transplantation.

### Pathology review

A total of 51 patients’ pathology slides were collected and rescored according to the Oxford Classification by central pathology review (S.S. Shi). Sections were stained with hematoxylin and eosin (H&E), Masson’s trichrome, periodic acid-Schiff (PAS), and PAS together with silver methenamine. The renal biopsies were reviewed by two pathologists using the Oxford Classification criteria independently and blindly to the clinical data. Mesangial hypercellularity (M) score of at least 0.5 is classified as M1; this score was the mean mesangial cellularity score in all scorable glomeruli based on the number of mesangial cells in the most cellular mesangial area, excluding the central core and region of the vascular pole (less than four cells, score of zero; four or five cells, score of one; six or seven cells, score of two; and eight or more cells, score of three). Endocapillary hypercellularity (E) and segmental glomerulosclerosis (S) were scored as absent (zero) or present (one). Interstitial fibrosis/tubular atrophy (T) was scored according to the estimated percentage of interstitial fibrosis and tubular atrophy in the cortex: T0 (≤25% of cortex), T1 (26%–50%), and T2 (>50%). Crescents (C) were scored according to the fractions of glomeruli with cellular or fibrocellular crescents: C0 (no crescent), C1 (<25%), and C2 (≥25%).

### Statistical analysis

Normally distributed variables, which are expressed as the mean ± SD, were compared using Student’s t-test. Nonparametric variables, which are expressed as medians and interquartile ranges, were compared using a Mann–Whitney U test or Fisher’s exact test if the number of patients with some variables was small. To analyze kidney survival from dialysis or for the composite kidney outcome, we used Kaplan–Meier survival curves and unadjusted Cox regression. In the present study, the follow-up of one patient was terminated due to all-cause death, and the time of death is unknown. Therefore, we estimated the follow-up duration according to the last laboratory test dates for this patient.

All *P* values were 2-tailed, and values <0.05 were considered statistically significant. Confidence intervals included 95% of predicted values. SPSS version 26 statistical software (IBM Corporation, Armonk, NY) and R programming software (version 4.1.3) were used for all analyses.

## Results

### Baseline clinical and pathological characteristics of excluded patients in the TESTING study from China

A total of 112 excluded patients from the TESTING study from 10 centers in China were enrolled in the present study, including 88 patients who were excluded due to proteinuria less than 1 g/24 h after receiving the maximum labeled or tolerated dose of RASB, along with optimized blood pressure control, and 24 patients with other reasons for exclusion, such as patient decisions or inappropriate eGFR.

In the present study, we mainly emphasized on the clinical and pathological characteristics of 88 patients who were excluded due to proteinuria below 1 g/24 h (Table 1). 43/88 patients (48.9%) were male. At the beginning of the run-in phase, the mean age was 37.8 years, and the 24-h urine protein excretion was 1.40 (1.11–1.82) g/24 h. The patients had a serum creatinine level of 109.7 ± 40.9 μmol/L, and the mean eGFR was 74.0 ± 29.6 mL/min per 1.73 m^2^. A total of 40 patients had available Oxford pathological scores, of whom 7/40 (17.5%) presented with M1, 8/40 (20.0%) with E1, 28/40 (70.0%) with S1, 14/40 (35.0%) with T1, 3/40 (7.8%) with T2, and 2/40 (5.0%) with C1, and no patients showed C2. The total 112 patients had similar clinical and pathological characteristics.

**Table 1.** Baseline clinical and pathological characteristics of patients excluded from the TESTING study.

	Overall (112)	Proteinuria < 1 g/24 h (88)	Other reasons (24)
Male (%)	53/112(47.3%)	43/88(48.9%)	10/24 (41.7%)
Age (years)	37.6 ± 10.2	37.8 ± 9.7	36.9 ± 11.7
Proteinuria (g/24 h)	1.43 (1.16–1.93)	1.40 (1.11–1.82)	1.71 (1.35–2.56)
Scr (μmol/L)	109.2 ± 40.9	109.7 ± 40.89	107.4 ± 42.0
eGFR (mL/min per 1.73 m^2^)	74.2 ± 29.0	74.0 ± 29.6	75.0 ± 27.6
MAP (mmHg)	98 ± 12	99 ± 12	96 ± 10
Pathology (%)			
M1	11/51 (21.6%)	7/40 (17.5%)	4/11 (36.4%)
E1	11/51 (21.6%)	8/40 (20.0%)	3/11 (27.3%)
S1	39/51 (76.5%)	28/40 (70.0%)	11/11 (100%)
T1, T2	18/51, 4/51 (35.3%, 7.8%)	14/40, 3/40 (35.0%, 7.5%)	4/11, 1/11 (36.4%, 9.1%)
C1	3/51 (5.9%)	2/40 (5.0%)	1/11 (9.1%)
Excluded due to proteinuria less than 1 g/24 h	88/112 (78.6%)		
RASB treatment after exclusion (%)	95/112 (84.8%)	73/88 (83%)	22/24 (91.7%)
Immunosuppressive therapy (%)	26/112 (23.2%)	16/88 (18.2%)	10/24 (41.7%)
Follow-up (months)	50.9 (26.4–83.4)	56.3 (8–101)	40.6 (26.4–84.2)
Proteinuria at last follow-up (g/24 h)	0.50 (0.22–1.08)	0.52 (0.20–1.06)	0.46 (0.28–1.79)
Scr at last follow-up (µmol/L)	102.1 (78.2–159.3)	108.0 (80.0–160.0)	95.0 (65.7–152.0)
eGFR at last follow-up (mL/min per 1.73 m^2^)	66.5 ± 36.3	64.6 ± 34.8	73.2 ± 41.7
Dialysis (%)	10/112 (8.9%)	7/88 (8%)	3/24 (12.5%)
All-cause death (%)	4/112 (3.6%)	2/88 (2.3%)	2/24 (8.3%)
Composite kidney endpoint (%)	14/84 (16.7%)	10/66 (15.2%)	4/18 (22.2%)

Abbreviations: proteinuria < 1 g/24 h, patients were excluded from the TESTING study due to proteinuria less than 1 g/24 h; Other reasons, patients were excluded from the TESTING study due to other reasons; Scr, serum creatine; eGFR, estimated glomerular filtration rate; MAP, mean arterial pressure; MESTC, MESTC scores by Oxford classification; RASB, renin-angiotensin system blocker.

Composite kidney endpoint, defined by *a* ≥ 50% reduction in eGFR, ESKD (eGFR <15 mL/min per 1.73 m^2^), chronic dialysis for at least 6 months or renal transplantation.

Overall, these 88 excluded patients were followed up with median of 56.3 (8.0–101) months. A total of 73/88 (83.0%) patients received RASB, and 16/88 (18.2%) received IST during the follow-up period after being excluded at the end of the run-in phase. 72/88 (81.8%) had stable proteinuria remission and did not receive IST. At the end of the follow-up, the mean proteinuria level was 0.52 (0.20–1.06) g/24 h, the mean serum creatinine level was 108.0 (80.0–160.0) μmol/L, and the mean eGFR was 64.6 ± 34.8 mL/min per 1.73 m^2^. Ultimately, 17/88 (8.0%) patients underwent dialysis, and 10/66 (15.2%) patients progressed to the kidney composite endpoint (defined by a reduction ≥50% in eGFR or ESKD). Two (2.3%) patients ultimately died from all causes (Table 1).

**Figure 2.**
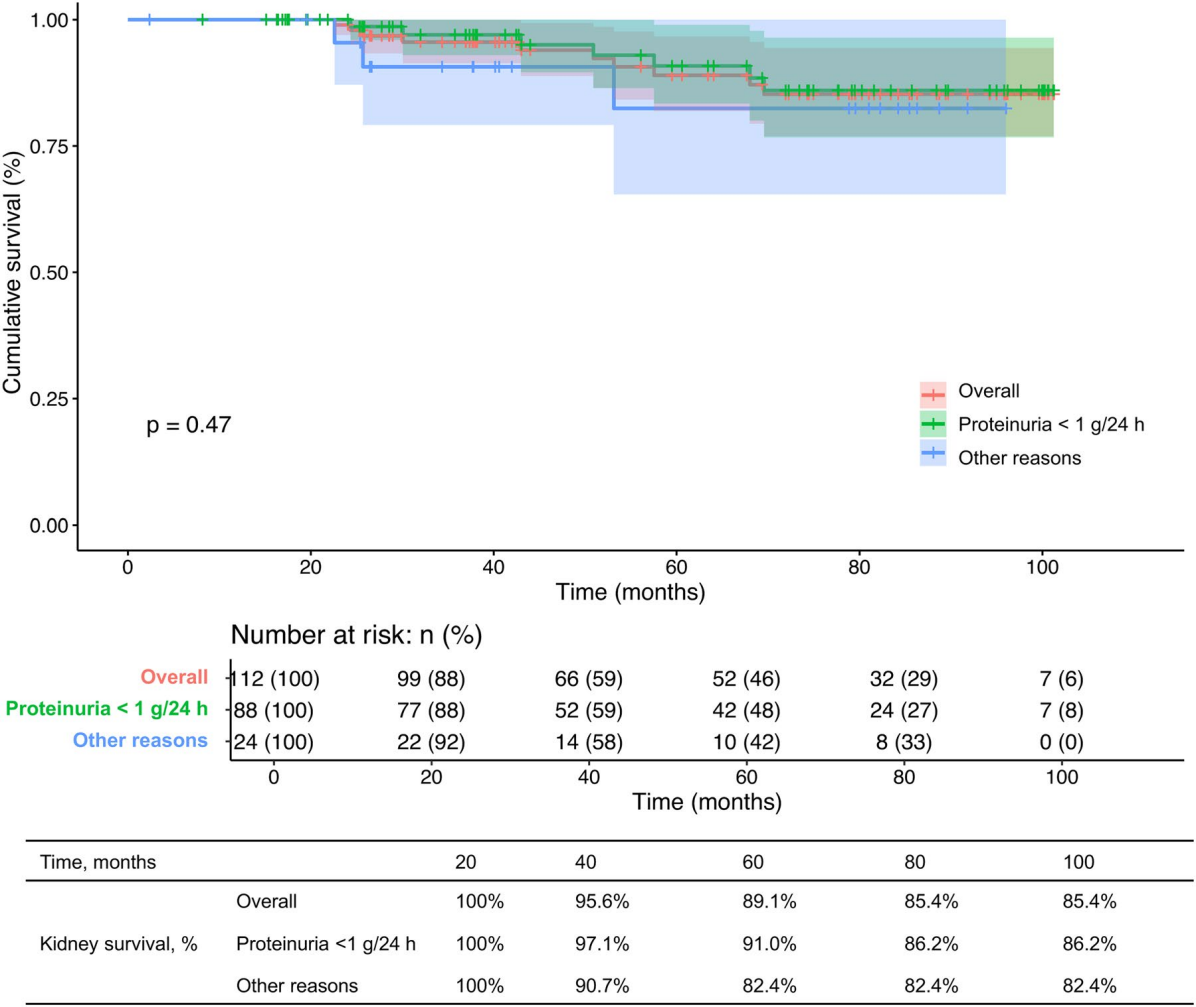
Kaplan–Meier curve analysis of kidney survival from dialysis among the overall patients, patients who were excluded from the TESTING study due to proteinuria less than 1 g/24 h, and patients who were excluded from the TESTING study due to other reasons.

The proportion of receiving IST during the follow up inpatients excluded due to proteinuria less than 1 g/24 h was significantly lower than that of patients excluded due to other reasons (16/88 (18.2%) vs. 10/24 (41.7%), *p* = 0.027). However, they had similar kidney survival from dialysis, as assessed by Kaplan–Meier analysis (*p* = 0.40). The 60-month and 100-month kidney survival proportions did not differ (60-month kidney survival: overall, 89.1%, proteinuria < 1 g/24 h, 91.0%, other reasons, 82.4%; 100-month kidney survival: overall, 85.4%, proteinuria < 1 g/24 h, 86.2%, other reasons, 82.4%) (Figure 2).

### Clinical characteristics and prognosis of patients excluded due to proteinuria less than 1 g/24 h

Among the 88 patients excluded due to proteinuria below 1 g/24 h, 72 had stable remission of proteinuria less than 1 g/24 h and did not receive IST during the subsequent follow-up. Among the 72 patients who had stable remission of proteinuria less than 1 g/24 h, 58 received RASB during the follow-up after exclusion, and 14 patients did not receive RASB. Patients who received RASB showed a better kidney outcome than those who did not receive RASB therapy, as well as lower proportions of progression to dialysis (5.2% vs. 14.3%, *p* = 0.229) and the composite kidney endpoint (11.4% vs. 25.0%, *p* = 0.299), respectively; however, these differences were not statistically significant.

16 patients received IST because of a relapse of proteinuria of more than 1 g/24 h. Among these 16 patients, 12 had relevant detailed information about the use of immunosuppressant. 2/12 patients received prednisone alone, 2/12 patients used prednisone and hydroxychloroquine together, 2/12 patients received Tripterygium glycosides alone, 1/12 patient received prednisone combined with Tripterygium glycosides, 1/12 patient used prednisone and mycophenolate mofetil together, 1/12 patient used prednisone and cyclophosphamide, and 1/12 used leflunomide. We further analyzed the difference in baseline characteristics and kidney prognosis between these two groups.

Patients with IST had more severe T1/2 lesions (100% vs. 36.1%, *p* < 0.001) at the time of kidney biopsy than those without IST therapy. These two groups had similar gender, MESC score at biopsy and similar age, baseline proteinuria, eGFR, MAP at the beginning of run-in phase in TESTING study. Among 16 patients received IST, 15/16 (93.8%) received RASB treatment together during the follow-up after exclusion. At the end of follow-up, patients with IST showed borderline significant severe proteinuria (1.08 g/24 h vs. 0.44 g/24 h, *p* = 0.078); however, patients with IST had similar eGFR values (54.5 mL/min per 1.73 m^2^ vs. 67.3 mL/min per 1.73 m^2^, *p* = 0.20), proportions of progression to dialysis (12.5% vs. 6.9%, *p* = 0.61) and composite kidney endpoints (18.8% vs. 9.7%, *p* = 0.38) compared with those not receiving IST among patients excluded due to proteinuria less than 1 g/24 h (Table 2).

**Table 2.** Baseline characteristics between patients exhibiting proteinuria less than 1 g/24 h with or without immunosuppressive therapy.

	Non-IST (72)	IST (16)	*P*
Male (%)	35/72(48.6%)	8/16(50%)	0.92
*Age (years)	38.5 ± 10.0	36.7 ± 12.0	0.19
*Proteinuria (g/24 h)	1.40 (1.10–1.84)	1.38 (1.20–2.42)	0.52
*Scr (μmol/L)	106.9 ± 38.0	123.7 ± 49.0	0.14
*eGFR (mL/min per 1.73 m^2^)	76.0 ± 29.6	64.1 ± 28.4	0.16
*MAP (mmHg)	99 ± 12	97 ± 11	0.41
Pathology (%)			
M1	6/36 (16.7%)	1/4 (25%)	0.68
E1	8/36 (22.2%)	0/4 (0%)	0.29
S1	25/36 (69.4%)	3/4 (75%)	0.82
T1, T2	12/36, 1/36 (33.3%, 2.8%)	2/4, 2/4 (50%, 50%)	0.003
C1	2/36 (5.6%)	0/4 (0%)	0.63
RASB treatment after exclusion (%)	58/72 (80.6%)	15/16 (93.8%)	0.29
IST after exclusion (%)	0/72 (0%)	16/16 (100%)	
Follow-up (months)	63.7 (26.3–87.7)	42.9 (26.2–65.6)	0.22
Proteinuria at last follow-up (g/24 h)	0.44 (0.20–0.90)	1.08 (0.38–1.78)	0.078
Scr at last follow-up (μmol/L)	102.1 (77.0–143.3)	127.0 (91.5–207.5)	0.12
eGFR at last follow-up (mL/min per 1.73 m^2^)	67.3 ± 35.8	54.5 ± 29.8	0.20
Dialysis (%)	5/72(6.9%)	2/16(12.5%)	0.61
Combined kidney endpoint (%)	7/72 (9.7%)	3/16 (18.8%)	0.38
All-cause death (%)	2/72(2.3%)	0/16(0%)	0.50

Abbreviations: No relapse of proteinuria, patients had a stable level of proteinuria less than 1 g/24 h; proteinuria relapse, patients with relapsed proteinuria more than 1 g/24 h; Scr, serum creatine; eGFR, estimated glomerular filtration rate; MAP, mean arterial pressure; MESTC, MESTC scores by Oxford classification; RASB, renin-angiotensin system blocker. IST, immunosuppressive therapy.

Composite kidney endpoint, defined by *a* ≥ 50% reduction in eGFR, ESKD (eGFR <15 mL/min per 1.73 m^2^), chronic dialysis for at least 6 months or renal transplantation.

* Data at the start of the run-in phase in TESTING study.

Because the follow-up time of both two groups varies, we selected the maximum follow-up time (100 months) that both groups could reach for our main analysis. By Kaplan–Meier curve analysis, the kidney survival from dialysis (Figure 3a, *p* = 0.23) and the composite kidney outcome (Figure 3b, *p* = 0.15) did not show a significant difference between excluded patients with or without IST. However, the 60-month and 100-month cumulative kidney survival proportions of the excluded patients in the IST group were significantly lower than those of the non-IST group (60-month kidney survival from dialysis: 83.3% vs. 91.7%; 60-month kidney survival for the composite kidney outcome: 64.3% vs. 85.8%; 100-month kidney survival from dialysis: 55.6% vs. 89.1%; 100-month kidney survival for the composite outcome: 32.1% vs. 72.1%).

Figure 3.(a) Kaplan–Meier curve analysis of kidney outcomes for chronic dialysis between patients with proteinuria less than 1 g/24 h who were receiving or not receiving immunosuppressive therapy. (b) Kaplan–Meier curve analysis of kidney outcomes for composite kidney endpoint between patients with proteinuria less than 1 g/24 h who were receiving or not receiving immunosuppressive therapy.

**Figure:**
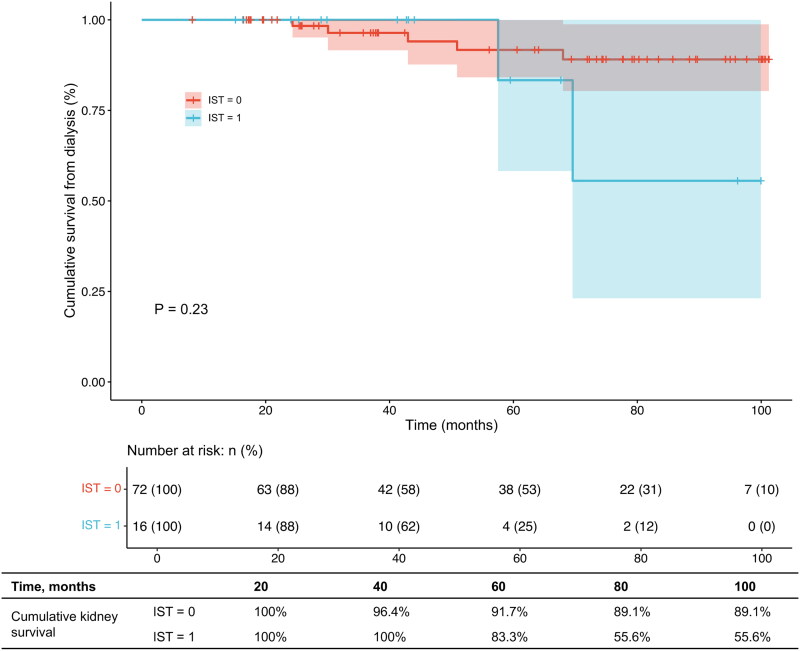

**Figure:**
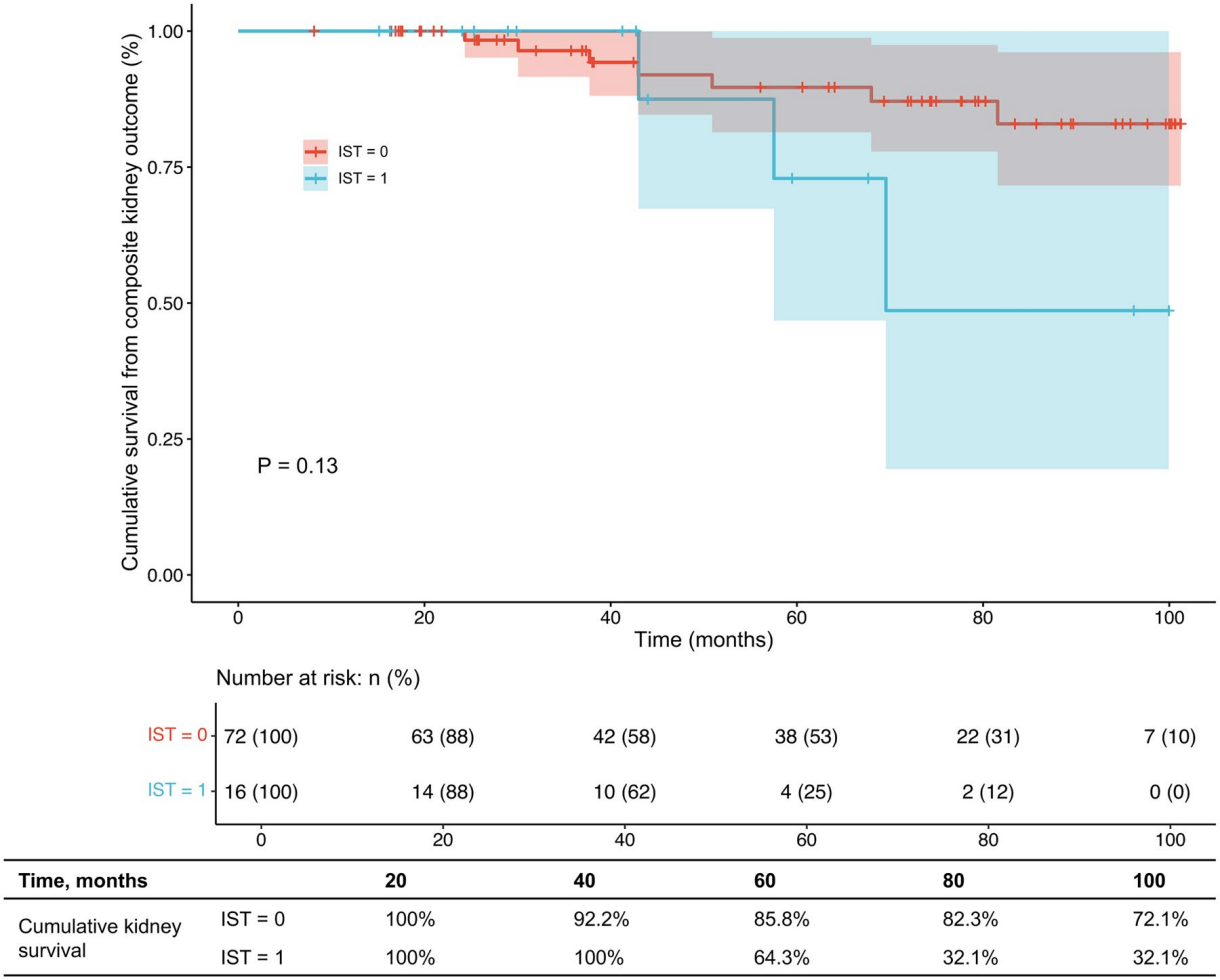

Moreover, the Kaplan–Meier analysis showed that no patients experienced dialysis before 57 months and a composite kidney endpoint before 40 months in the IST group, which may have affected the statistical significance of the overall curve. Considering of the clinical significance, we selected 60 months and 48 months as cutoffs for dialysis and composite kidney outcome separately. Therefore, we further performed landmark analysis of kidney outcomes between two groups. Before 60 months, the kidney survival from dialysis of excluded patients with IST was similar to that of excluded patients without IST (*p* = 0.788); however, after 60 months of follow-up, the risk of progression to dialysis of excluded patients with IST was significantly higher than that of excluded patients without IST (OR = 11.3, *p* < 0.03) (Figure 4a). Similarly, before 48 months, excluded patients with IST had a similar risk for the composite kidney outcome to those without IST (*p* = 0.862), but after 48 months of follow-up, the risk of progression to the composite kidney outcome was significantly higher in excluded patients receiving IST than in those not receiving IST (OR = 5.92, *p* = 0.029) (Figure 4b).

Figure 4.(a) Landmark analysis of kidney outcomes for chronic dialysis between patients with or without immunosuppressive therapy. (b) Landmark analysis of kidney outcomes for composite kidney endpoint between patients with or without immunosuppressive therapy.

**Figure:**
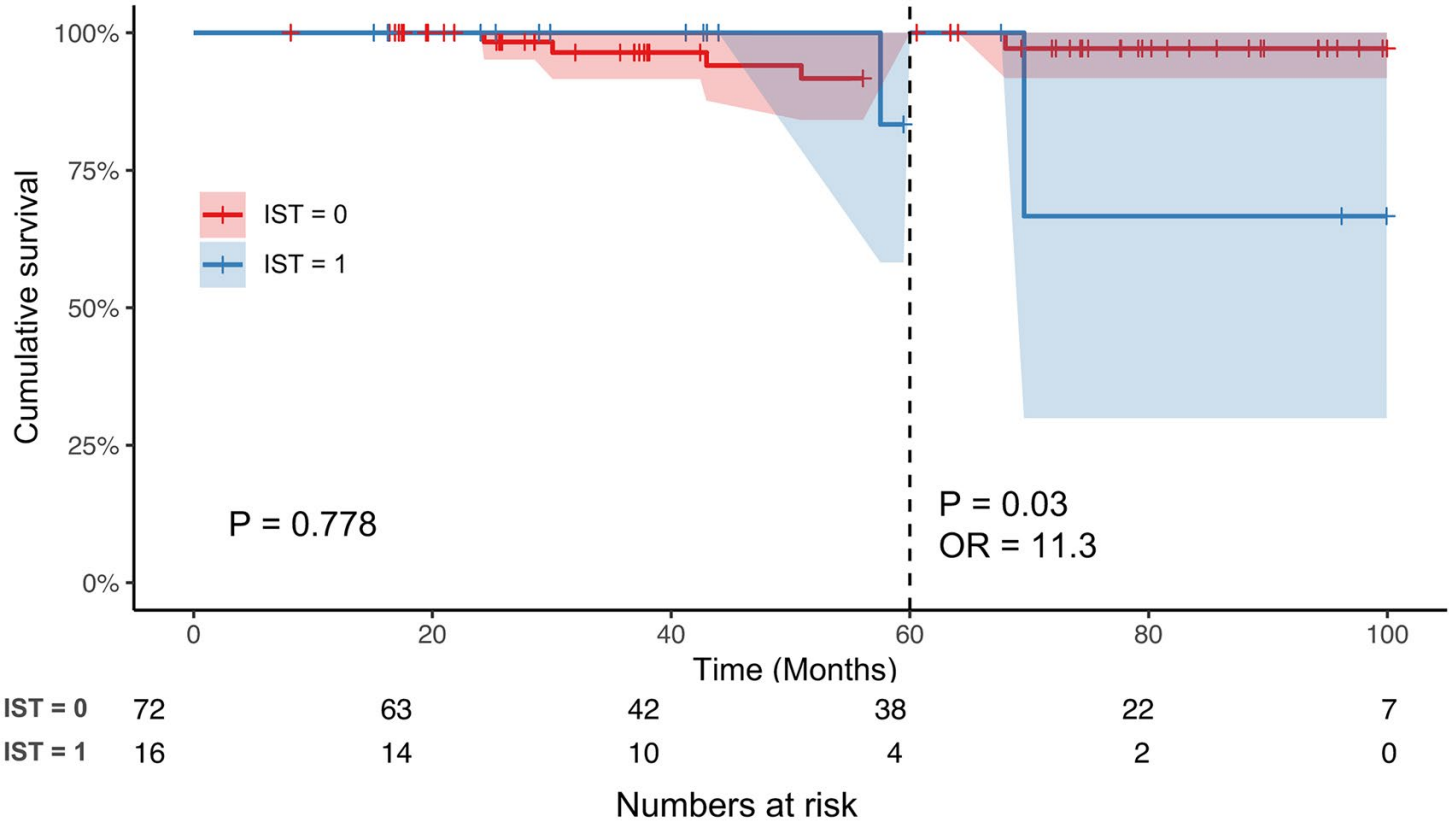

**Figure:**
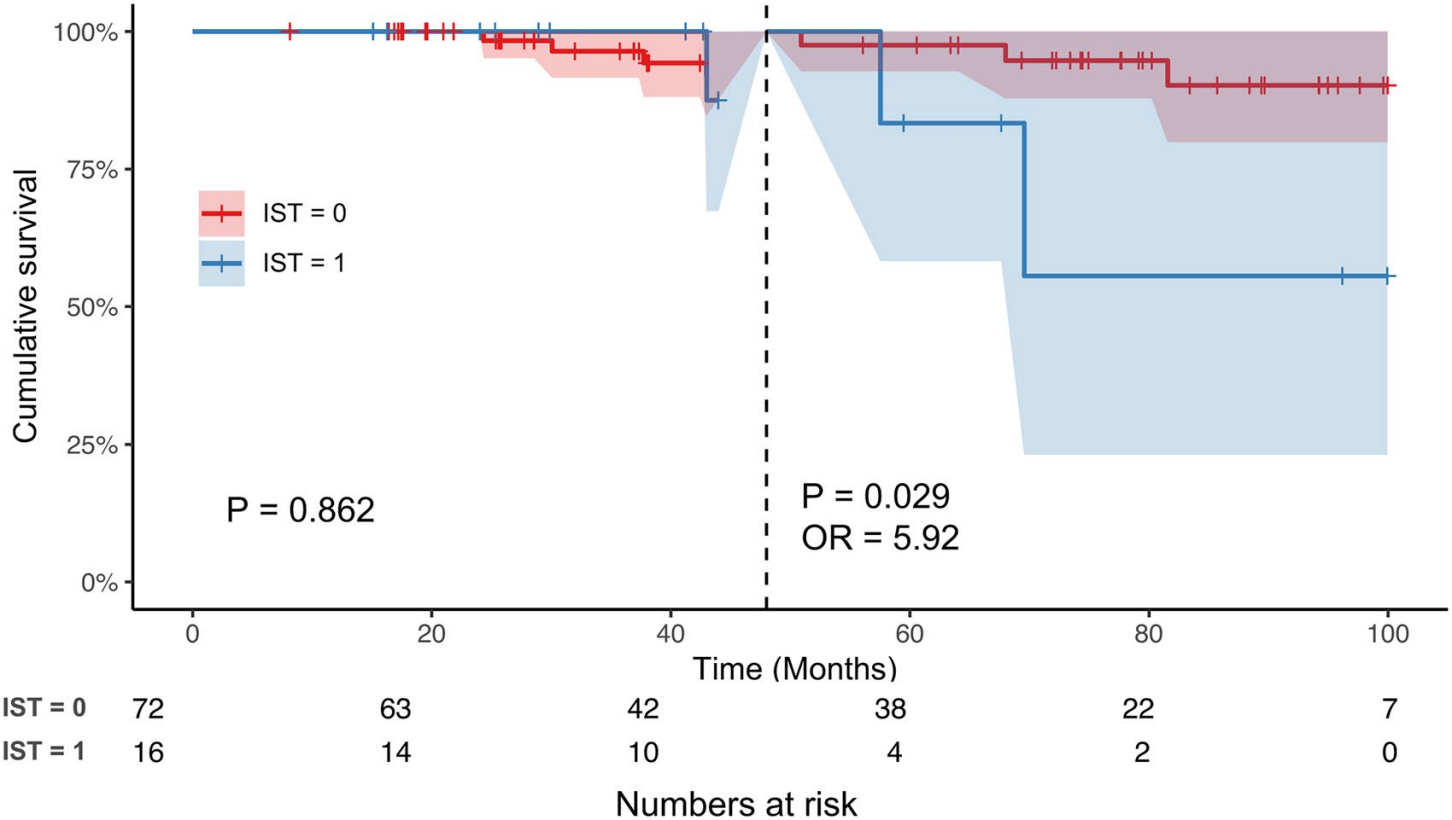

### Prognostic factors for kidney outcome in patients with proteinuria less than 1 g/24 h

We further analyzed the prognostic factors for kidney outcome in the patients excluded due to a low level of proteinuria less than 1 g/24 h. Using univariate Cox analysis, only worse baseline kidney function and T lesions were risk factors affecting the prognosis for kidney outcomes from dialysis (eGFR, HR 0.95, 95% CI, 0.91–0.99, *p* = 0.026; T1/2 lesions, HR 7.77, 95% CI, 2.04–29.7, *p* = 0.003). However, by multivariate Cox analysis, the predictive value of eGFR and T1/2 lesions for the kidney outcome was not statistically significant.

## Discussion

The TESTING study confirmed the benefit of oral corticosteroids for patients with IgAN whose proteinuria remained greater than 1 g/24 h and kidney function was suitable after 12 weeks of intensive supportive treatment with RASB. However, the clinicopathological characteristics and prognosis of patients excluded from the TESTING study, especially those excluded due to proteinuria less than 1 g/24 h after intensive supportive care, were still unclear. In the present study, we found that the long-term kidney prognosis of IgAN patients who were excluded due to proteinuria less than 1 g/24 h after intensive supportive therapy was good when they had persistent remission of proteinuria and no need for steroid therapy.

It is well known that proteinuria is a strong and independent predictor of long-term kidney outcomes [3,5,12,13]. In the present study, IgAN patients who had been excluded from the TESTING study because of proteinuria remission from 10 centers in China were enrolled. We found that among the 88 patients whose proteinuria could be remitted by intensive supportive treatment, 16 patients experienced relapse of proteinuria, which was defined as a level greater than 1 g/24 h. Although the patients who experienced a relapse of proteinuria recurrence had similar level of baseline proteinuria to those without relapse, they had a worse baseline kidney function and more severe T lesions. In a prospective clinical trial, Li Y et al. also found that IgAN patients whose proteinuria remained between 1 and 3.5 g/24 h after ACEI/ARB for at least 90 days and received prednisone therapy, the remission percentage in those without T1/T2 was significantly greater than the subgroup with T1/T2. However, because of the limitation of the sample size, especially with the limited pathology data, we could not identify factors that could predict proteinuria relapse and a need for IST after proteinuria remission achieved by intensive supportive therapy. Moreover, even though all 16 patients received immunosuppressive therapy, their long-term kidney prognosis was worse than that of patients without proteinuria relapse. Although there have been many studies on the association between the decreased degree of proteinuria and the prognosis of IgAN, studies on the duration of proteinuria remission are very limited. Reich et al. [3] found that patients who reached < 1 g/24 h proteinuria regardless of their starting point had a good kidney prognosis, which was similar to that of patients whose proteinuria never exceeded 1 g/24 h, whether the remission was reached spontaneously or with intervention. In a multiethnic retrospective study by Canney et al. that included 1864 IgAN patients [14], virtually any duration of proteinuria reduction conferred a clinical benefit to patients with IgAN, and the longer an individual maintained proteinuria remission, the better their long-term outcome. This result was consistent with our landmark analysis, which also demonstrated that once patients reached remission of proteinuria after intensive supportive therapy, even patients in the relapsed group still had a short-term kidney outcome that was as good as that in the non-relapsed group (within 60 months for dialysis and 48 months for the composite kidney outcome). However, with the prolonged follow-up time, the risk of dialysis or composite kidney outcome for patients in the relapsed group was respectively 11.3 times or 5.92 times higher than those in the non-relapsed group.

There is much evidence about the early activation of the renin-angiotensin system (RAS) in the kidneys of IgAN patients [15,16], which is associated with the pathogenesis of tubulointerstitial damage [17]. The clinical benefits of RASB in kidney diseases have been proven in several IgAN clinical trials and is considered one of the major pillars of supportive treatment [7,8,18,19]. In the present study, among the 72 patients who had stable remission of proteinuria less than 1 g/24 h, 58 received RASB during the follow-up after exclusion, and 14 patients did not receive RASB. We found that those who received RASB treatment during the subsequent follow-up after being excluded had clinical and pathological characteristics that were similar to those in patients who did not receive RASB; however, patients who received RASB showed a better kidney outcome than those who did not receive RASB therapy, as well as lower proportions of progression to dialysis (5.2% vs.14.3%, *p* = 0.229) and the composite kidney endpoint (11.4% vs. 25.0%, *p* = 0.299), respectively; however, these differences were not statistically significant, which might be because of the small sample size of the present study. These results suggested that long-term RASB therapy should be provided even when proteinuria remission has been achieved.

The present study has some limitations. First, this is a retrospective cohort study, and we did not have enough information during the follow-up to investigate the factors that could indicate a flare of proteinuria and the need for steroid therapy. Data at the time of started IS therapy, as well as the adverse events and effectiveness such as proteinuria remission or eGFR decline during IS therapy, were not collected. Second, only part of patients who were excluded from the TESTING study in China because of proteinuria less than 1 g per day were enrolled, so the sample size was small. From our results, most of these patients with proteinuria maintained less than 1 g/24 h and did not progress to ESKD, and due to the widespread use of steroids therapy in China, there is no group with proteinuria above 1 g per day that was not treated with IS. Third, not all enrolled patients had Oxford pathology data. In addition, since these patients were not followed up regularly, some patients did not adhere to taking RASB consistently after exclusion; moreover, the reason why some patients used IST therapy, the detailed dosage and the side effect of IST was not recorded, and we could not obtain the time-averaged proteinuria. A larger sample of research is needed to confirm the results and to identify factors predicting proteinuria relapse.

In conclusion, our study suggested that IgAN patients who maintained a persistent remission of proteinuria after intensive RASB therapy had a much better long-term kidney outcome than those who experienced a relapse of proteinuria.
